# Supplementary material for: Bmapaf-1 is Involved in the Response against BmNPV Infection by the Mitochondrial Apoptosis Pathway
Source: Insects. 2020 Sep 22;11(9):647. doi: 10.3390/insects11090647 (PMC7563707; doi:10.3390/insects11090647)
Supplement: Supplementary file 1 [file insects-11-00647-s001.pdf]

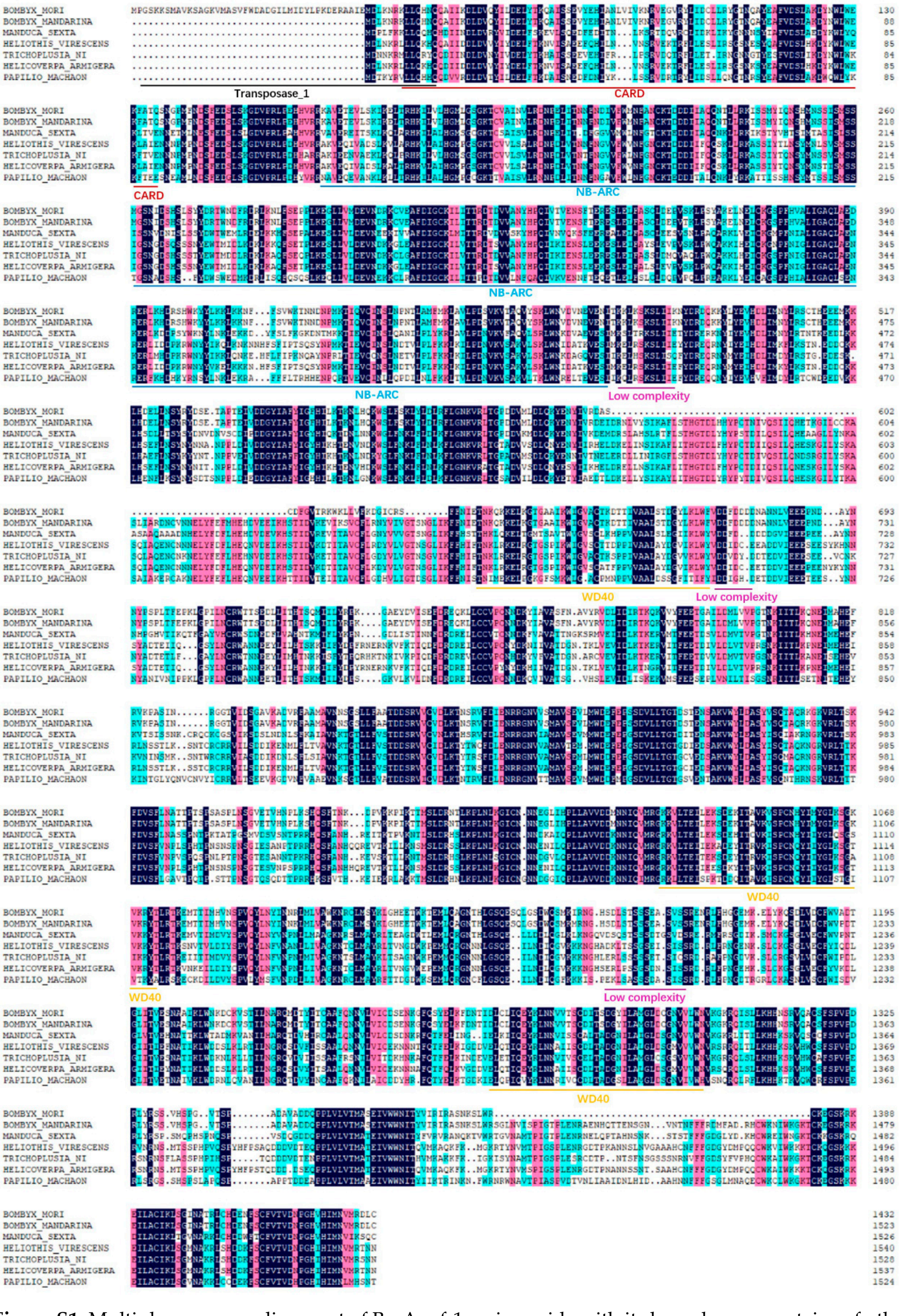

Figure S1. Multiple sequence alignment of BmApaf-1 amino acids with its homologous proteins of other species. The deduced amino acid sequence of BmApaf-1 and its homologs in other species were retrieved from the NCBI database. Identical amino acids are highlighted in dark blue, and the positive amino acids are highlighted in pink and aqua.

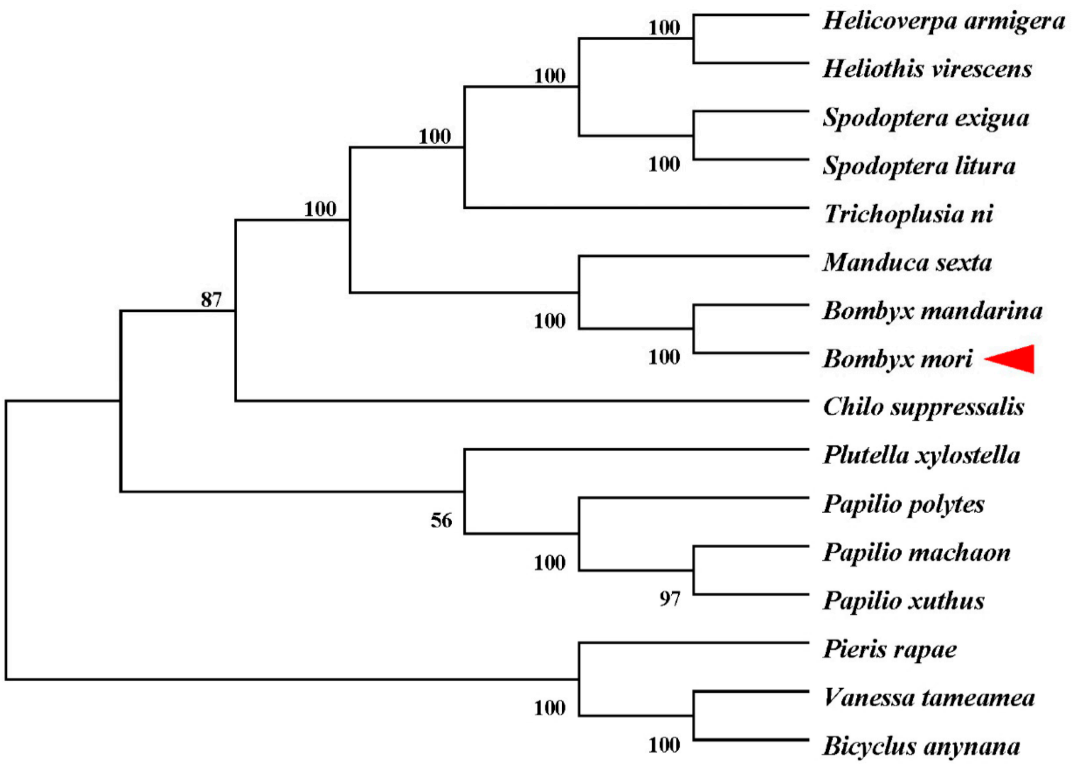

Figure S2. Phylogenetic analysis of BmApaf-1 using the neighbor-joining method. The tree was constructed with pairwise deletion of gaps in MEGA-X. The percentages on the branches indicate bootstrap values from 1000 replicates. The tree is drawn to scale, with branch lengths in the same units as those of the evolutionary distances used to infer the phylogenetic tree. The evolutionary distances were computed using the *p*-distance method. The analysis was included 16 amino acid sequences. All positions containing gaps and missing data were eliminated. Taxonomic names are shown on the right of the branches.

Table S1. The list of accession numbers of proteins using in phylogenetic analysis

Species	Accession numbers
Bicyclus anynana	XP_023937388.1
Bombyx mandarina	XP_028036457.1
Bombyx mori	NP_001186937.1
Chilo suppressalis	RVE48846.1
Helicoverpa armigera	XP_021181657.1
Heliothis virescens	PCG76501.1
Hyposmocoma kahamanoa	XP_026319294.1
Manduca sexta	XP_030022507.1
Papilio Machaon	XP_014359375.1
Papilio polytes	XP_013138265.1
Papilio Xuthus	KPI94114.1
Pieris rapae	XP_022124006.1
Plutella xylostella	AHB86312.1
Spodoptera exigua	AYM00395.1
Spodoptera litura	XP_022828360.1

Trichoplusia ni	XP_026734098.1
Vanessa tameamea	XP_026496270.1
